# Semiquantitative Risk Evaluation Reveals Drivers of African Swine Fever Virus Transmission in Smallholder Pig Farms and Gaps in Biosecurity, Tanzania

**DOI:** 10.1155/2024/4929141

**Published:** 2024-05-13

**Authors:** Folorunso O. Fasina, Niwael Mtui-Malamsha, Hezron E. Nonga, Svetlana Ranga, Rosamystica M. Sambu, Jerome Majaliwa, Enos Kamani, Sam Okuthe, Fredrick Kivaria, Charles Bebay, Mary-Louise Penrith

**Affiliations:** ^1^Food and Agriculture Organization of the United Nations, Rome, Italy; ^2^Department of Veterinary Tropical Diseases, University of Pretoria, Onderstepoort 0110, South Africa; ^3^Food and Agriculture Organization of the United Nations, Lusaka, Zambia; ^4^Ministry of Livestock and Fisheries, Dodoma, Tanzania; ^5^Tanzania Veterinary Laboratory Agency, Temeke, Dar es Salaam, Tanzania; ^6^Food and Agriculture Organization of the United Nations, Nairobi, Kenya

## Abstract

African swine fever (ASF) has remained persistent in Tanzania since the early 2000s. Between 2020 and 2021, pig farms in twelve districts in Tanzania were infected with ASF, and ≥4,804 pigs reportedly died directly due to the disease with disruption to livelihoods. We conducted semiquantitative field investigations and rapid risk assessment (RRA) to understand the risk factors and drivers of ASF virus (ASFV) amplification and transmission in smallholder pig farms, and determine the gaps in biosecurity through hazard profiling, focus group discussions and expert opinion. Outbreaks were connected by road and aligned along the pig product value chain and reported in the northern, central, and southern parts of Tanzania. The patterns of outbreaks and impacts differed among districts, but cases of ASF appeared to be self-limiting following significant mortality of pigs in farms. Movement of infected pigs, movement of contaminated pig products, and fomites associated with service providers, vehicles, and equipment, as well as the inadvertent risks associated with movements of animal health practitioners, visitors, and scavengers were the riskiest pathways to introduce ASFV into smallholder pig farms. Identified drivers and facilitators of risk of ASFV infection in smallholder pig farms were traders in whole pigs, middlemen, pig farmers, transporters, unauthorized animal health service providers, and traders in pork. All identified pig groups were susceptible to ASFV, particularly shared adult boars, pregnant and lactating sows, and other adult females. The risk of ASF for smallholder pig farms in Tanzania remains very high based on a systematic risk classification. The majority of the farms had poor biosecurity and no single farm implemented all identified biosecurity measures. Risky practices and breaches of biosecurity in the pig value chain in Tanzania are profit driven and are extremely difficult to change. Behavioural change communication must target identified drivers of infections, attitudes, and practices.

## 1. Introduction

**Figure 1:**
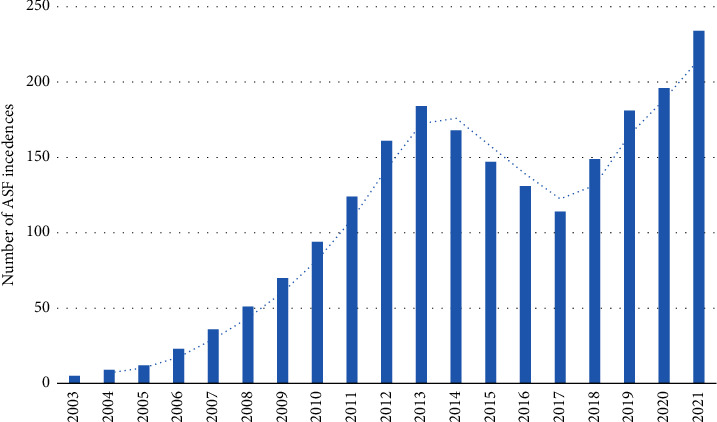
Modelled epidemic curve of African swine fever introduction and transmission in the United Republic of Tanzania, 2003–2021, based on pattern of reporting. Phase I: incursion (from 2003 to 2008), Rt = 0.5 ≤ *N* ≥ 0.8, sporadic events in the rural areas (Katavi and Mikumi areas); phase II: establishment/persistence (from 2009 to 2016), Rt = 1.3 ≤ *N* ≥ 1.7, persisting epizootics in the more urban areas (Kinondoni); phase III: dispersion (from 2017 to 2021 and continuing), Rt = 1.7 ≤ *N* ≥ 2.6, new territories are now infected. Based on seasonality, the Rt ≥ 2 for the period between November and February of the following year. ^*∗*^Note that the 2021 outbreak is continuing and the cumulative number of cases may exceed what was documented in the graph above.

African swine fever (ASF) remains an important pig disease globally in view of its rapid spread, economic impacts, and food security implications. There is no treatment, and although a vaccine against a genotype II Vietnamese field strain has recently been developed [1] and is registered for limited use, mainly in Asia, further evaluation is needed and efficacy against multiple strains of several genotypes is unlikely. Tanzania probably has a long history of ASF because in the first published description of ASF [2], in addition to the outbreaks in settlers' pigs in Kenya, the author reported receiving a letter from the Chief Veterinary Officer of Tanzania (then German East Africa) describing two outbreaks of what he believed to be ASF in pigs in that country. Tanzania was also the first country in which ASF was reported from ticks of the *Ornithodoros moubata* complex collected in animal burrows frequented by warthogs [3]. Confirmed ASF in domestic pigs was first reported in Tanzania in 1962 [4] and since that period, outbreaks have continued to occur in Tanzania periodically. However, from the early 2000s, it appears that the frequency and intensity of ASF occurrence in Tanzania has increased considerably (Figure 1), and many foci of regular infections have been established in the major pig-producing areas of Tanzania. In particular, since 2010, the occurrence of ASF has taken a dramatic turn following a major outbreak that started in Kyela, Mbeya region, followed by another that occurred in 2012 [4, 5]. In 2013, an unrelated focus of the disease was detected in the northern Highland region of Kilimanjaro, which later spread to the Arusha region. Since that period, ASF has more or less persisted as an endemic disease with annual cycles of infection in Tanzania.

**Figure 2:**
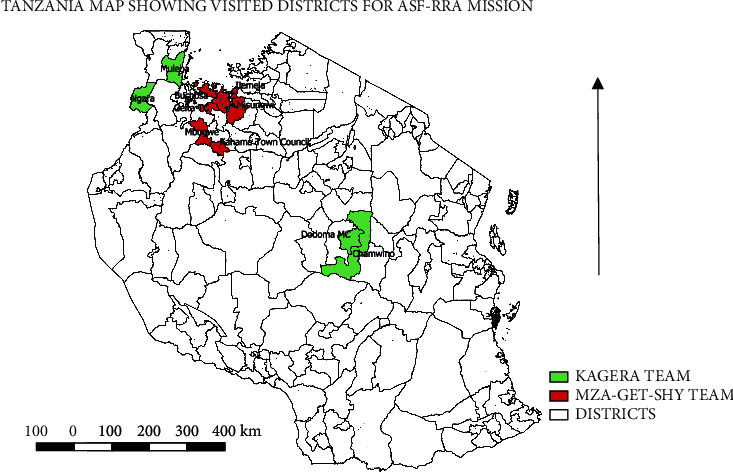
Map of Tanzania showing the areas visited during the ASF RRA. The Kagera team covered Kagera (Ngara and Muleba districts) in the extreme northwest part of Tanzania and Dodoma (Dodoma Jiji and Chamwino districts) regions in the central area. The MZA-GET-SHY team covered Mwanza (Sengerema and Mwanza Jiji districts), Geita (Geita district council), and Shinyanga (Mbogwe and Kahama districts) regions in northwest Tanzania. A total of 5 regions and 9 districts were covered. Each team consisted of a national epidemiologist, a zonal veterinary officer, zonal laboratory personnel, the district veterinary officer of the affected district, and relevant field officers.

Based on retrospective analysis and traceback data, the outbreak of ASF in 2020-2021 started with some sporadic cases of ASF in the Katoro ward of Geita District Council in June 2020. This spread to Geita Town council by July 2020 and was officially notified to the National Epidemiological Unit of the Directorate of Veterinary Services, Ministry of Livestock and Fisheries (DVS-MoLF). The disease later spread to the neighbouring districts and municipalities of Mbogwe, DC, Sengerema, and Kahama Municipal Councils (Figure 2).

There was a period of quiescence between July and early November 2020, and whether this was due to the absence of cases or no reports from the farmers was not established. The disease was again reported in early November 2020. In total, between March 2020 and March 2021, twelve Local Government Authorities in the regions of Mwanza, Geita, Shinyanga, Kagera, Dodoma, Mbeya, and Iringa reported outbreaks (Table 1 and Figure 2). At least 4,804 pigs were reported to have died directly due to the disease in the affected regions by 29^th^ March 2021 in Tanzania (Table 1 and Figure 2). Two teams or at least five professionals (epidemiologist, zonal veterinary officer, zonal laboratory personnel, district veterinary officer, and pathologist/animal handlers) visited the infected five regions and nine districts.

Initial supports to the subnational animal health authorities included intensive awareness campaigns using risk communication and community engagement (RCCE) strategies and laboratory diagnosis. Additional support was provided to the Government of the United Republic of Tanzania by the Food and Agriculture Organization (FAO) of the United Nations, including support to conduct additional field investigations, rapid risk assessment (RRA), data collection for socioeconomic evaluation, and scaled up RCCE. The objective of the current study is to investigate the plausible risk factors for and drivers of farm infection and outbreaks of African swine fever in order to inform prevention and control strategies.

## 2. Materials and Methods

### 2.1. Approach to the Risk Assessment

To conduct the comprehensive risk assessment for the ASF epidemiological situation in high-risk districts of the country and identify the plausible risk factors and drivers that amplified or facilitated the transmission of the ongoing outbreaks in the study area, a triaging process was undertaken with a threshold of 7/14 score to justify or negate the follow-up field activities (Supplementary Figure 1 and Supplementary Table 1) [6]. In addition, the teams were established, hazard was profiled, risk questions were formulated, data collection and literature review were conducted, and the process was reviewed through expert opinion as per the established protocol for rapid risk assessment [6].

### 2.2. Description of the Study Area

#### 2.2.1. Field Data Collection, Sampling, and Laboratory Analysis

Using a snowballing method, field data collection was done using qualitative participatory approaches including key informants (KIIs, *n* = 45), focus group discussions (FGDs, *n* = 34), visualization/observations and transect walking with stakeholders in the value chain to mark the farm biosecurity checklist, and expert knowledge elicitation (EKE, *n* = 19). The key informants are veterinary doctors (*n* = 7), veterinary paraprofessionals (*n* = 2), individual farmers (*n* = 32), butchers (*n* = 2), one transporter, and one district executive director, who have worked directly with the pig subsector in the regions concerned for at least five years. FGDs were held with 18 mixed professional groups and 16 single professional groups (farmers, *n* = 9; butchers, *n* = 4; livestock field officers, *n* = 2; and vendors, *n* = 1). In total, 224 individuals were involved and distributed as follow: farmers (*n* = 101), middlemen (transporters and traders, *n* = 19), butchers (*n* = 43), ward executives (*n* = 11), village executives (*n* = 3), extension officer (*n* = 1), rural development officers (*n* = 4), livestock field officers (*n* = 20), veterinary officers (*n* = 21), and District Executive Director (*n* = 1). A combination of semistructured interviews and predetermined checklists were utilized for data gathering. An exhaustive literature review was also conducted to explore missing data that are available from relevant peer-reviewed publications from PubMed, Google Scholar, and available grey literature.

Furthermore, secondary data on timelines of outbreaks, mortalities, and estimated cost implications were obtained from the district veterinary officers (DVOs) of the infected districts. Through the KII and FGD, risk pathways were developed, including the identified risk factors (hazard (pathogen), release/entry, and exposure/transmission of the pathogen) in the outbreak areas. During the field data collection, biological samples (spleen, mesenteric, and gastro-hepatic lymph nodes) from dead pigs or from stored samples at the Zonal Veterinary Centres were obtained and dispatched to the African swine fever (ASF) laboratory at the Sokoine University of Agriculture (SUA), Morogoro, or the Tanzania Veterinary Laboratory Agency, Centre for Infectious Diseases and Biotechnology (TVLA-CIDB), Temeke, Dar es Salaam, Tanzania. ASF laboratory diagnosis was based on the partial amplification and sequencing of p72 nucleotides of ASF virus (ASFV) as previously described [7, 8].

#### 2.2.2. Self-Rated Biosecurity Assessment

For assessment among smallholder pig farmers, biosecurity, which refers to measures taken to minimize the risk of introduction of new pathogens into or outside the farm premises, was grouped under three elements, viz. segregation, cleaning, and disinfection [9]. Biosecurity consists of (1) bioexclusion, which involves preventing the introduction of new pathogens into a farm animal population from an outside source; (2) biocontainment, which involves preventing the inadvertent release of pathogens present in the farm from other farms and facilities; and (3) biomanagement, which involves managing pathogens that are present in the farm to minimize negative consequences. Bioexclusion measures include fencing the premises with documented entry via controlled gates, introduction of new animals from guaranteed safe sources or quarantine of new animals for at least 14 days, ensuring safety of feed and water, limiting access to the pigs and providing at least dedicated footwear for necessary visitors, or ensuring the use of well-managed disinfectant footbaths. On larger commercial farms, measures may include showers, quarantine, barns, inlet filters where feasible, decontamination and changing rooms, feed supply from outside the farm alone over the fence, and measures to ensure safe transportation. Biocontainment involves controlled exit of pigs, people, and products, farm-level best practices in terms of waste management, and disposal of dead pigs, as well as transparent disease reporting and ensuring that inadvertent release of pathogens into other farms and facilities does not occur via people or fomites. Biomanagement involves cleaning and disinfection of the pig premises, avoiding overstocking, and all-in-all-out systems in larger farms with observation of downtime. Within this context, a 25-item biosecurity checklist was prepared based on biosecurity measures protective against ASF with potential for uptake among smallholder and small commercial-confined production. This checklist was used to capture farm-level compliance on biosecurity measures (Supplementary Table 2).

#### 2.2.3. Expert Opinion/Expert Knowledge Elicitations

Based on the subjective but specific information (data, facts, arbitrary and anecdotal information, etc.) gathered from the value chain actors, an expert knowledge elicitation (EKE) was used to validate the knowledge and provide additional judgements (probabilities, estimates, etc.) based on experts' experience. To implement EKE, a total of 25 people were invited through an independent e-mail (facilitated Delphi survey), but 19 experts responded (76% response rate), including 12 national and seven international experts from the field of infectious diseases, virology, epidemiology, animal health, border vigilance, and epidemio-surveillance. In this study, an expert is defined as a person with at least five years of field or clinical experience related to African swine fever or significant peer-reviewed publications in the field of African swine fever (the list of experts is available on request). A set of six questions was provided to this pool of national and international experts through an e-mail post. The list of variables that formed the questions originated from issues previously identified by the stakeholders during the KII and FGD. Responses to questions 1–3 were provided through a rank order scaling system, while questions 4 and 5 were through a Likert scale score (1–10) (Supplementary Tables 3–6; Supplementary Figures 2 and 3). For question 6, a pool of opinions was provided to the expert, who could add or remove opinions with empirical reasons for the decision. Each expert independently responded to the questionnaire. All responses were entered into Microsoft Excel 2016® spreadsheet for filtering and analysis. To reduce the effects of experts' personal and subjective views and own beliefs, Delphi opinion survey analysis was conducted through consensus or mathematical aggregation of experts' estimates until a general agreement was reached after two rounds of surveys. Mean scores obtained were utilized to triangulate the original information gathered through field data collection.

#### 2.2.4. Identification of Hazard and Its Potential Pathways

Based on the qualitative risk assessment method, all potential risk contributors, drivers, and factors were identified and listed. The pathways of exposure and transmission were mapped with local field experts, and the estimates and risk levels were done using the experts' opinions. The categories of risk for the introduction of ASF to smallholder pig farms were fomites to pigs, feed to pigs, byproducts to pig, and pig to pig. The warthog to pig, ticks to pig, as well as environment to pig, were the lowest ranking factors. In northern Europe, wild boars, which are ancestral to domestic pigs, die of ASF and their carcasses contaminate the environment. If these carcasses are not rapidly removed, it helps to keep the infection going. Warthogs and other African wild suids are resistant to the effects of the virus and do not die of ASF or contain infective amounts of ASFV, so do not contaminate the environment [2]. The tick-to-pig cycles are either related to warthogs or to ticks that live in pig sties (Supplementary Figure 3). Plausible measures aimed at risk reduction were identified for each of the pathways (Supplementary Table 6). The risk analysis was based on the broad pathways evaluated during the mission for the possible entry of the disease into Tanzania, dissemination within the country, and further spread to contiguous countries.

Furthermore, using ASF as the current hazard, the risk of introduction, intra- and interfarm transmission (spread) of African swine fever within Tanzania's smallholder farms and from across the border were classified through experts' consensus opinions. In addition, the risk of hazard among the groups of pigs in the farm (adult shared boars, nonshared boars, pregnant sows, other adult females, growers, workers, weaners, and piglets) was compared.

#### 2.2.5. Statistical Analysis

Descriptive statistics and other analyses were conducted using Microsoft Excel 2016® spreadsheet. In brief, described variables were listed in the order in which they appeared on the questionnaire. Ranks, as provided by the experts were listed against the ordered variables. Frequency tables were created to determine the number of times each variable was ranked from 1 to 15), and the rank frequencies were determined using the function “ = COUNTIF($Range, $Criteria)” on the spreadsheet. The sum of rank frequencies was determined for the rows and columns using the function “ = SUM(Range).” This process was repeated for each variable. On a separate sheet, tabulation was made by transposing the variables on the column to the ranks on the rows. Using the transposed results, the total scores for the ranks were obtained by calculating the total points for each row (rank 1*∗*15 + rank 2*∗*14 + rank 3*∗*13 ………….. rank 15*∗*1). The final mean rank for all experts was obtained by using the function “ = RANK(deposition,$Range) (https://www.youtube.com/watch? v = eza1XbeD2Hc).

For questions 4-5, inter-rater agreements between foreign and local experts' scores were calculated using the modified method of Landis and Koch [10] and Beck et al. [11]. In brief, mean scores of local and international experts were obtained for each question, and the disparity from the full score was calculated (e.g., 10.0−9.33 = 0.67). Using the online Cohen's Kappa calculator (https://www.graphpad.com/quickcalcs/kappa1/?*K* = 3), the mean scores and the disparity from full scores were entered into the appropriate cells to generate the Kappa scores, standard errors, and 95% confidence intervals.

### 2.3. Ethical Approval and Consent to Participate

This study was fully approved by the Government of Tanzania under the project code GCP/GLO/074/USA. No handling of animals beyond normal outbreak investigation activities, which do not require ethical approval, was involved, and the study did not use sensitive or protected data. All experimental protocols were approved by the Review Committee of the Ministry of Livestock and Fisheries, Tanzania, with the approval number MA 154/355/16. All methods were carried out according to the relevant guidelines and regulations [12]. All participants gave informed consent and willingly signed the consent register independently. They were also informed of their right to withdraw participation at any stage of the study.

## 3. Results

In total, we conducted 45 key informant interviews, 34 focus group discussions, and 19 expert opinion elicitations, covering five regions and nine districts. The key issues that arose from the discussions include the following.

### 3.1. Spatiotemporal and Epidemiological Data

Based on our field mission and discussions with stakeholders involved in the pig and pig products' value chain in Tanzania, ASF was reported in 12 district councils between March 2020 and March 2021 (Figure 2 and Table 1). The majority of the outbreak locations was connected by road and is aligned along the pig and pig products' value chain in Tanzania. Outbreaks in the lake zone were reported from Sengerema DC, Geita DC, Geita TC, Mbogwe, and Kahama Municipal Councils. In addition, the districts of Ngara, Muleba, Chamwino, and Dodoma municipalities had reported outbreaks (Table 1). Other district or municipal councils with outbreaks but not covered in the current investigation include Mpwapwa, Kyerwa, Misungwi, Mbeya, Busokelo, and Iringa, mainly in the southern axis of Tanzania. The patterns of outbreaks and impacts differed from one district to the other, with some wards reporting up to 100% mortality and over 80% of the pig farming households directly affected by ASF through farm-to-farm infections. All clinical pathological samples (*n* = 12) submitted to the laboratory were confirmed by PCR using the p72 gene of ASFV. In the visited district councils, cases and deaths associated with ASF appeared self-limiting following significant mortality of pigs in farms or emergency sales of apparently healthy pigs to reduce farm-level losses. Using the historical outbreak reports (2003–2013) to the model from 2014 to 2021, our modelled estimated number of outbreaks was in excess of 230 farm-level infections (Figure 1). At the time of compiling this outbreak report (16^th^ May 2021), a total of 79 farm-level infections had been reported. The national and subnational veterinary authorities put a quarantine in place during the active outbreaks, but such quarantines were lifted in batches in selected districts following the end of outbreaks and no report of a new case for a period of at least 28 days. Furthermore, the urban and periurban districts have higher numbers of animal health officers compared to the more rural districts (Table 1).

### 3.2. Hazard Classification, Risk Pathways, Risk Factors, Contributors, and Drivers

**Figure 3:**
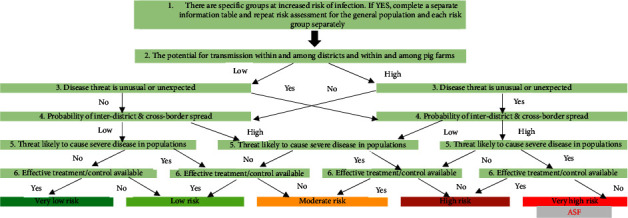
Risk classification for African swine fever virus (hazard) among Tanzania's smallholder pig farms, 2021. Mean agreement score ±standard deviation = 9.6 ± 0.7; median = 10 (1 = strongly disagree to 10 = strongly agreed). Kappa ± SE = 0.90 ± 0.10 (95% confidence interval: 0.88–0.92); the interrater agreement between foreign and local experts' scores were calculated using the method of Landis and Koch [9]. Adapted from European centre for disease prevention and control. Operational guidance on rapid risk assessment methodology [21]. This figure was drawn based on the RRA questions in annex 7. Based on the focus group discussions and key informant interviews, the risk was particularly high among pregnant sows, adult female, shared adult boars, nonshared boars, porkers, growers and less among weaners and piglets in that order. In most cases, survivors are the young animals (piglets and weaners), and piglets may die due to cessation of milk when the sow dies.

Based on stakeholder identification and experts' opinions, the movement of infected pigs, movement of contaminated pig products, and fomites associated with service providers, vehicles, and equipment, as well as the inadvertent risks associated with movements of animal health practitioners, visitors and scavengers were the riskiest pathways to introduce ASFV into smallholder pig farms in Tanzania (Table 2 and Supplementary Table 7). Similarly, laboratory personnel, arthropods (flies, including Stomoxys and ticks), infected live pigs imported through formal routes, and manure and bedding were the least likely risk pathways to introduce ASFV into smallholder pig farms in Tanzania (Table 2 and Supplementary Table 7). In terms of drivers and facilitators of risk of ASFV infection in smallholder pig farms, traders in whole pigs, middlemen, pig farmers, transporters, unauthorized animal health service providers, and traders in pork were the most important drivers and facilitators identified (Table 3 and Supplementary Table 8). The least risky facilitators included feed manufacturers, wild pig hunters, police and other law enforcement officers, and border control officers. Finally, based on experts' opinions, the risk of ASFV is relatively high in all farmed pig groups but is particularly high among shared adult boars, pregnant and lactating sows and other adult females, and nonshared adult boars and growers. Weaners, piglets, and porkers are less likely to be infected but may nevertheless die from indirect consequences of ASF in the herd. For instance, it was reported that piglets often die due to starvation associated with the death of their dams (Table 4 and Supplementary Table 9). Overall, using stakeholder consultations (KII and FGD) and experts' opinions, the risk of ASF for smallholder pig farms in Tanzania was considered very high based on a systematic risk classification (Figure 3). The mean agreement score was 9.6 ± 0.7, and the Kappa interrater agreement score was 0.90 ± 0.10 (95% CI: 0.88–0.92).

### 3.3. Farm-Level Biosecurity Evaluation

**Figure 4:**
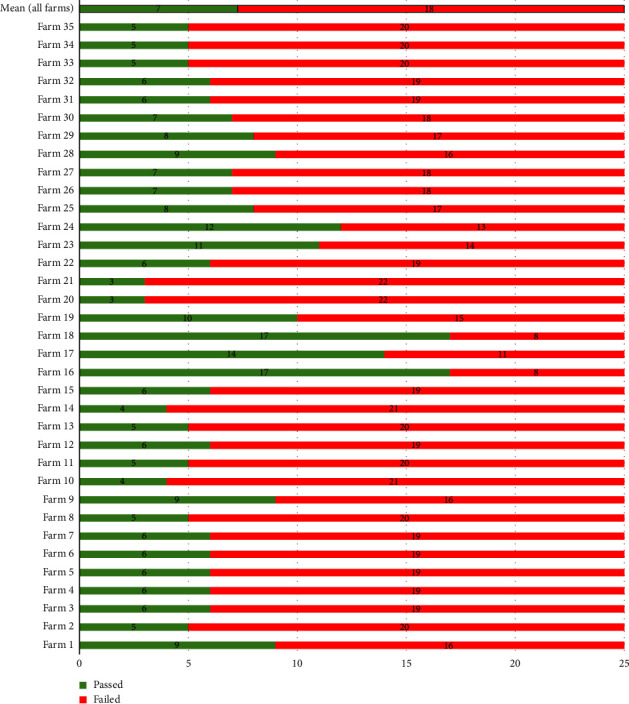
Biosecurity scores for smallholder pig farms using a 25-measure point scale, Tanzania (mean value for all farms with bold edges).

Based on the 25-item biosecurity checklist, 82.9% (29/35) of all respondents' farms implemented up to 10 of the items listed, 8.6% (3/35) implemented between 11 and 15 items, and only 5.7% (2/35) implemented 17 out of the 25-item checklist (Figure 4). No single farm implemented all 25 measures in the pig farm. While these scores are based on a checklist, observations and in-depth queries during the KII and FGD raised questions on whether the measures were applied regularly (constantly) or just periodically (episodically). Using the farm-level evaluation of the 25-item score on specific biosecurity measures implemented in 35 pig farms, the mean score for all premises evaluated was 29% ((*n* = 7/25 measures) (min = 12% (*n* = 3), median = 24% (*n* = 6), maximum = 68% (*n* = 17)). It is noteworthy that only two farms implemented 17 of the 25 biosecurity measures and only three farms exceeded 50% of all biosecurity measures implemented. These findings have implications for increased infection risk for animal diseases (Figure 4).

## 4. Discussion

### 4.1. Disease Reporting, Epidemio-surveillance, Value Chain, and Implications for Amplification of Interfarm, Interdistrict, and National Spread of ASF in Tanzania

Disease emergencies are immediately reported by farmers to the field extension officer (EO), agricultural officer (AO), or livestock field officer (LFO) or where these are not available, to the village or ward executive officers (VEOs or WEOs), who in turn report to the responsible district veterinary officer (DVO). The shortfalls in the number of staff needed at the district level sometimes have implications for the effectiveness of delivery of animal health services including reporting and timely response (Table 1). Sometimes, the delayed reporting by farmers increased the intensity and impact of the outbreaks, resulting in higher morbidity and mortality. The plausible explanations for this delay include inadequate staffing at the ward level, poor knowledge regarding the disease by field staff, resorting to self-help by farmers, as well as poor knowledge of biosecurity, hazard, and its transmission pathways among the farmers. For instance, in some districts under investigation, farmers indicated that they had administered pen-strep, tylosin, or other antibiotics and only reported later to the official authorities when no positive response was obtained. Often, on receipt of reports, the DVOs conduct clinicopathologic examination, report to the Directorate of Veterinary Services, and liaise with the relevant zonal veterinary centres and zonal veterinary laboratory under the TVLA for sample collection. These facilities have competent manpower and medium-level resources for sampling but may not be effective for confirmatory diagnosis of ASF, often due to lack of reagents and consumables. With the introduction of the Event Mobile Application (EMA-i) in over 60% of LGAs in Tanzania, the quantity and quality of animal disease reports have improved as the DVOs can interconnect and undertake near real-time reporting electronically. In the current evaluation, approximately 66.7% of the districts under investigation have submitted recent ASF reports through EMA-i applications within weeks of outbreak.

Regarding awareness, the field officers, pig farmers, butchers/traders, and many of the stakeholders could clearly describe ASF clinical signs, a useful attribute for epidemio-surveillance. However, the knowledge of infection routes and transmission varied widely among work groups, being best among veterinarians and animal health/livestock field officers, medium to high among the extension and agricultural officers, medium among farmers, traders, and butchers, but medium to poor among the ward and village executive officers [14, 15].

Interdistrict coordination of movement restrictions during outbreaks is often lacking among contiguous districts and regions. For example, when quarantine was imposed in an infected district during a period of intense outbreaks, it was initially in the affected wards only. However, because the compliance level was poor, such quarantines were often extended to cover the whole district, yet interdistrict movements sometimes occurred from noninfected districts into infected districts and vice versa. Typically, noninfected districts have no quarantine imposition and no border vigilance, primarily because of the overstretched workforce. Similarly, intra- and interdistrict, multidistrict, and international cross-border movements, particularly to large livestock markets and slaughter slabs, are reported by the DVOs in charge in each district (Supplementary Figures 4–6).

The smallholder farmers sourced their pigs from within their immediate wards, districts, regions, or from distant districts. Some are purchased directly from nearby livestock markets or from traders who purchase pigs for slaughter. These farmers, particularly those who live in border towns and villages, sometimes source pigs from Burundi, Rwanda, Uganda, and Kenya but also from Zambia, Malawi, and Mozambique.

An official movement and import permit system exists, but farmers and traders sometimes evade the official system by moving pigs and pig products across intranational and international borders during the night and at odd hours. Due to the extensive stretch of Tanzania's borders, it is difficult to perform effectively police movements or carry out effective vigilance and surveillance duties, given the limitations in available manpower in the government system. In addition, stakeholders most often do not seek professional guidance ahead of purchase, and traders and butchers sometimes intentionally or inadvertently buy infected pigs, which are sold much more cheaply (at between 20 and 50% of the normal trade value). Similar observations have been reported previously from Vietnam [16]. Furthermore, there is a tendency for traders/butchers to source pigs for slaughter from Burundi and Rwanda but may instead sell them to farmers, and neither these traders nor farmers isolate the new arrivals.

### 4.2. Identified Drivers and Risk Factors

#### 4.2.1. Upstream Water Source

A good number of smallholder farmers depend on water from streams as drinking water for their pigs and for washing the pig houses/pens and equipment, and the runoff goes back to the stream. This consequently regularly contaminates the stream and increases the risk of ASF if any of the farms along the stream are infected. This phenomenon was clearly demonstrated in Sengerema as well as Geita, where following ASF infection in the upstream farms, the ASF infection spread down streams and affected other pig farms. Similar observations were made previously in the southern highlands through contamination from slaughter slabs upstream, which later flow downstream towards Lake Nyanza [5]. In some areas, such as Mabatini in Mwanza, and in city centres such as Dodoma, smallholder farmers have abandoned the use of water from streams and rather utilize piped or well water, following infections from the previous outbreaks of 2017. Anecdotal evidence pointed to a causation between contaminated waters and ASF infection of pig farms; the possibility of carcasses being thrown into streams, thus creating heavy contamination and a source of infection cannot be excluded. A high oral dose is needed to produce infections in pigs, which is plausible for streams with low volumes of water, which are receiving a lot of runoff from farms and slaughter slabs. On large rivers, the source of infection is more likely to be carcasses that wash up on the banks and are feasted upon by scavenging pigs. In our evaluation, most commercial farmers constructed and use boreholes and treated water for their farms.

#### 4.2.2. Slaughter Slabs/Areas

No appropriate handling and slaughter facilities are available to pig farmers and traders and neither has any standard design been constructed as a proof of concept for the farmers and other stakeholders. In addition, because many communities have a significant number of Muslim populations, pig slaughter facilities cannot be combined with those for other livestock species, and such facilities must be constructed in societally acceptable locations. Given the foregoing constraints, pigs are slaughtered mostly in poor, unhygienic or decrepit slaughter slabs, often located within pig farms, or in some distant location. Some stakeholders have made personal efforts to improve the standard of the slaughter slabs, but these facilities still lack the necessary equipment and tools expected for a standard abattoir facility. Pigs slaughtered for these slabs come from various sources within the different districts, or from other districts, and as far as from outside the country. When animals are imported illegally, efforts are made by farmers and traders to mix them with the owned stock within the farms to evade confiscation and destruction of the untested imported pigs. Most of these slaughter slabs lack disposal pits and are not fenced, hence easily accessed by dogs and free-roaming pigs.

#### 4.2.3. Selected Farm Management Practices

- Sharing of boars
- Most farmers tend to hire/borrow boars from fellow farmers during breeding. A farmer in one of the investigated districts hypothesized that her sows were probably infected from the neighbouring farm. Following the dispatch of her sow to the other farm for mating and the observation of pig deaths in the other farm, she retrieved her sow and returned it to her farm. In total, she lost 14 sows, 7 growers, and 44 piglets and had only six survivors left. Similarly, Muleba alone experienced 101 ASF outbreaks. Following traceback, it was concluded that the outbreak in Muleba started from a ward where a farmer brought in a boar from another district for genetic diversity and improving his productivity. The imported boar became morbid and was slaughtered and the meat was shared in the community. Thereafter, disseminated outbreaks of ASF were reported in many wards in Muleba. At least 24 of the interviewed persons identified the practice of sharing of boar as a high-risk activity that contributes significantly to spreading ASF in pig farms.
- Unrestricted inflow and outflow of people/animals with lack of biosecurity measures
- Many pig farms lack adequate fencing or are unfenced, making them easily accessible to visitors, persons with malicious intentions of infecting pigs intentionally [15], scavenging animals (dogs, cats, and rodents), or stray pigs. In addition, pig traders and farm-gate buyers move from one piggery to another and from pen to pen when selecting potential pigs to purchase for redistribution or for slaughter. Sometimes, these farm-gate buyers, traders, and butchers, who operate without observing biosecurity protocols, inadvertently serve as sources of infection through intradistrict pig mobility, carriage of infected material on their shoes, clothes, knives or other tools/vehicles, or in the process of multisourcing of pigs from farm to farm. In addition, interdistrict and transboundary movements of people and pigs have high potential to result in inadvertent infection of farms with ASFV. Furthermore, hardly any footbaths, change of clothing, and use of gumboots were observed (Figure 4). Because most of the buildings on smallholder farms were constructed with wooden materials or scrap metal with a concrete or earthen floor, thorough cleaning and disinfection were very difficult, resulting in conditions favourable for a buildup of pathogens and consequent exposure of pigs to infections.
- Waste disposal
- None of the farms visited had a standard waste disposal pit for infected carcasses. In addition, no slurry pit was sighted for the collection of solid waste mixed with liquid and these just flow freely in the gutters outside the pigpen. It was observed that many of the farmers contaminate the environment by throwing the manure over the side of the pen or over the fence. Such practices also attract more scavengers and rodents into the farm premises, thereby increasing the risk of introduction of animal diseases.
- Humans as virus spreaders (animal attendants, farm managers, farm owners, paraveterinarian, and veterinarians)
- In most smallholder farms, the farm attendants, typically one per farm, are hardly trained in good farm management practices that should provide efficient pig management and welfare to the pigs but are expected to gather experience during their duties. These attendants serve all pig pens and, in most cases, do not observe biosecurity protocols such as the systematic movement from young to older animals or other biosecurity measures. During an on-farm study carried out on a medium-sized pig farm in Uganda where an outbreak of ASF had occurred, breaches of security when measures were in place were frequent and included failure to wear protective clothing provided and misplaced and incorrectly used disinfectant footbaths [17]. This underlines the principle that having a biosecurity plan is only successful if it is implemented consistently by everyone involved. When this is not the case, the risk of random introduction of animal diseases and transmitting them within the farm is high. In addition, farm managers and owners often used their positions to invite visitors to their farms. These visitors are in most cases persons with interests in animal farming and have high potential for interfarm introduction of diseases. Farm-gate buyers, traders, and butchers are also invited by farm managers to select pigs as mentioned above. During epizootics and animal health crises like the widespread outbreaks of ASF, para-veterinarians and veterinarians are often invited to provide animal health services. Due to the shortage of these categories of workers in the periurban and rural areas, as well as the shortfall of resources, these individuals often have to move from farm to farm, reusing instruments and using disinfectant sparingly if at all and may inadvertently transmit infection to new premises. Human activities (anthropogenic factors) have been identified as critical to the long-distance jumps of ASF introduction to new premises [5, 14, 16, 18]. For instance, the transport of contaminated meat or meat products, which may end up as waste or kitchen leftovers for feeding pigs, the purchase and introduction of untested boars, the transboundary informal purchase of new pigs, and subsequent mixing with the local stock in order to evade confiscation all pose a high risk of introduction of ASFV.
- Farm-level and community-level biosecurity
- Based on our evaluation, implementation of the 25 identified biosecurity measures was very low. It should be understood that a breach in the biosecurity protocols, particularly at a period when the farm is at a high risk of infection or in an overwhelming epidemic situation with a lot of acute cases, can eliminate all gains from hard work put into biosecurity implementation before the breach.

#### 4.2.4. Transnational, Cross-Border, and Country-Level Risks of ASF Entry, Reintroduction, and Exposure

Pig movement across the United Republic of Tanzania is random and diffuse, and long-distance animal movements are observed along the primary and secondary roads [5]. Within-village movements are done by trekking the pigs on foot or on bicycles. However, other forms of movement rely on motorcycles, tricycles, and motor vehicles (small vehicles and trucks). In this study, we clearly identified five patterns of movement for pig and pig products, including the following:

1. Interward/intervillage movements within a district.
2. Interdistrict and transdistrict movements across contiguous or distant districts, and from region to region.
3. Transboundary movements across national borders, particularly to Burundi and Rwanda in the north and from Zambia, Malawi, and Mozambique to the south but also from Uganda and Kenya.
4. Farm ⟶ open market ⟶ abattoir/slaughter slab ⟶ Farm.
5. Farm ⟶ Farm.

The value chain marketing and trade systems closely drive these pig and pig products movements with many forms of sale practices, i.e., formal, informal, farm-gate, and random types. These movement patterns have large implications for disease introduction and transmission. Traders, marketers, and farm-gate buyers move among farms without observing any biosecurity measures. They often move with their potentially contaminated tools, knives, shoes, clothes, vehicles, and restraining materials. In a few instances, movements were formal, with pigs and their products subjected to physical clinical examination and or laboratory testing, but the largely informal movements utilized unscrupulous means to evade veterinary authority detection by clandestine movement through unpatrolled border areas to smuggle pig products across national and international borders. A knowledgeable key informant from Burundi (through a phone call across the border) is indicated as follows:

*“ASF will never stop circulating in the subregion unless a regional approach to tackling the disease is implemented*.*”*

He confirmed that cheaper pigs, which are usually available during outbreaks, are traded across borders freely and there are not enough officials to manage intranational and cross-border animal health, surveillance, border vigilance, and disease control along the extensive borders. Hence, the cross-border and country-level risks of ASF entry, reintroduction, and exposure from neighbouring countries remain very high.

Almost all farm management practices listed are anthropogenic factors since they are human driven. Similar factors were recently reported in Uganda [15]. In other instances, humans act directly as vectors of the virus, hence intensifying risk communication and community engagement to encourage behavioural change targeting the identified anthropogenic factors should reduce the burden of ASF [15]. The ongoing transmission of the warthog-tick sylvatic cycle and the tick-to-pig transmission cycle, which are occurring elsewhere and which cannot be linked to any specific human practice or activity, are examples of nonanthropogenic transmission that are uncommon or documented in Tanzania. However, like the large jumps of ASF in Europe from distant infected wild boar populations to uninfected wild boars hundreds or thousands of km away, the situation observed in the current outbreaks in Tanzania is definitely anthropogenic and trade/movement-mediated [19].

#### 4.2.5. Consequences of the Outbreak and Its Socioeconomic Importance to the Pig Industry

Among the individual stakeholders interviewed, the majority (86%) had experienced ASF in their herds between June 2020 and February 2021; most of the respondents have lost between ≤95% and 100% of their stock due to ASF. The salvaged pigs were sold rapidly or slaughtered to recover approximately 25–30% of the normal market value; sometimes, the young ones (piglets and weaners) recovered and were kept as replacement stock. There are deficiencies in the knowledge of transmission, mitigation measures, and application of biosecurity in order to reduce the risk of infection. The farmers have lost businesses including (1) the loss of supply of pork to a niche market in the mining sector, (2) loss of major sources of income and livelihood, and (3) loss of food security and ability to support the family by providing funds for school/college fees and hospital bills and construction in the homes. Narrating his experience, a farmer stated as follows:

*“The children are back from school/college for the Easter break and I am disturbed and heartbroken; my pens are empty and I have lost everything. In total, I have lost as much as TSh 80 million (≈US$ 34,500) based on the scale of my operations*.*”*

A mission farm, which is supporting a popular community health program through the money accrued from the sales of live pigs and pig products and which supplied breeding stock to smallholder farmers and reached ≈1,000 farm families in remote settings of Ngara, lost approximately 98% of the herd. Another farmer lost over 400 pigs, and in another instance, a farmer withdrew two children from educational facilities (one at university and another in secondary school). Stakeholders, particularly farmers, were sentimental and expressed negative emotions against the authorities, whom they perceived to have left the stakeholders to their woes. The government will need to consider a reorganization of the pig farming system and the associated value chain in order to mitigate the risks associated with ASF.

#### 4.2.6. Information and Knowledge Gaps

Due to the shortage of animal health staff, agriculture officers (AOs), extension officers (EOs), ward executive officers (WEOs), and village executive officers (VEOs) sometimes perform double roles of routine administration and issuing animal movement permits and attending to other animal health matters. Farmers indicated that such officers sometimes promote the use of penicillin-streptomycin, sulphur-based antimicrobials, tylosin, and multivitamins for the treatment of high fever. In addition, the border control staff and officers certify animals crossing the official borders while collecting revenues for the government. The FGD and KII revealed that many of such officers were untrained in matters of animal health despite being tasked with the responsibility of issuing movement permits. A number of these officials interviewed could not identify enough clinical signs, symptoms, and pathological details associated with ASF and could not list the risk factors or facilitators of transmission. There is a need to develop a training package customized for the needs of lay officials (nonanimal health professionals) providing animal health services.

In addition, the knowledgeable farmers only gained sufficient knowledge based on their own farm experiences of pig farm infections. Many are, however, unaware that neither treatment nor vaccines are available for ASF and of the specific risk factors and benefits of biosecurity in mitigating ASF risk. Traders confirmed that they prefer to continue to buy low-priced pigs even though they acknowledge that it may contribute to spreading the disease, which may damage the pig industry. Their motivation was to enhance the profit margin. The butchers similarly slaughtered infected pigs, purchased at takeaway prices from desperate farmers during ongoing outbreaks of ASF, thereby contributing to the spread of the disease.

## 5. Conclusion

Risky practices and breaches of biosecurity in the pig value chain in Tanzania are profit driven and, therefore, extremely difficult to change. Aliro and colleagues [15] have reached a similar conclusion on why the Ugandan farmers failed to prevent and control ASF in their herds including the following reasons: (1) due to costs associated with biosecurity implementation, (2) the need to prioritise family livelihood over disease transmission risks, and (3) the incompatibility of current biosecurity practices with local culture, traditions, and social contexts and the lack of access to veterinarians or low-quality veterinary services. The shortfalls in subnational staff quantity have implications on the effectiveness of delivery of animal health services and epidemio-surveillance. The poor biosecurity scores have practical implications for increased infection risks for animal diseases at the farm level and the current pig and pig-products marketing and trade systems, and associated movement are direct drivers for disease introduction and transmission of pathogens. [20].

## 6. Recommendations

Based on the evaluation conducted, it is recommended that

- The relevant authorities should consider designing and building prototype dedicated and biosecure pig slaughter slabs that reduce environmental contamination. The siting of such slaughter slabs should be decided through a consultative process taking note of sociocultural as well as religious considerations of the community and guidance from the environmental authorities.
- Enforceable by laws should be in place to forbid homestead or farm-directed movement of adult pigs meant for abattoirs or slaughter slabs.
- The knowledgeable animal health officers, especially the DVOs and the LFOs, should develop scheduled timetables for the training of farmers, traders, and other stakeholders on biosecurity, good farming practices, movement, and marketing networks that minimise the risk of infection and transmission of ASF. Resource allocation to support such training should be made available from the revenues and fees generated from animal resources within the districts or region, with support from the national government and other stakeholders.
- The need for training and retraining of regional, district, and border officials involved in animal health services cannot be overemphasised. The training should focus particularly on emergency preparedness and response, as well as disease reporting. This should prevent delays in reporting animal health emergencies at the district or regional level and facilitate coordination with the central veterinary system.
- The issue of inadequate staffing, particularly in more rural districts, should be prioritised and addressed. This has earlier been identified in the findings of the 2016 Joint External Evaluation (JEE) in Tanzania [13]. It should include compiling a comprehensive inventory of animal health personnel in the country to determine the personnel gaps at the national, regional, and district levels.
- The delivery of effective animal health services at the district level needs adequate mobilization to respond promptly. The lack of mobility (motorcycles, vehicles, and bicycles), identified by the district level officers, must be addressed in a phased approach.
- A comprehensive animal resources evaluation at the district, regional, and national levels in Tanzania must be undertaken to compile a comprehensive animal inventory, determine the total economic value, and identify the inapparent opportunities to enable the government to generate revenues internally, some of which can be utilised to provide for the needs of animal health services at the subnational level.

The authorities may consider setting up pig demonstration and training farms and breeding centres in strategic districts/regions in the country. Such centres should be used to provide training on the pig value chain, integrated farm-level biosecurity, good husbandry practices, and good management practices (GMPs) [21, 22]. It should be understood, however, that training on biosecurity practices may positively affect gains in knowledge, but it may have little or no effect on farmers' attitudes and practices [14]. In this study, despite the intensive training on biosecurity, farmers would still allow veterinarians who may not have practiced biosecurity measures on their farms, even during outbreaks, would not restrict visitors from farm visits, be unlikely to deny traders access to the farms and less educated farmers are still likely to sell pigs during ASF outbreaks [14]. The causes of this are multifactorial and need to be explored through engagement with farmers and other stakeholders [23–25]. In the long term, the progressive reorganization of the livestock industry to align with the Tanzania Livestock Modernization Initiative and Tanzania Livestock Master Plan is imperative [21, 26].

## Figures and Tables

**Table 1:** Districts and regions reporting outbreaks and covered during the field investigation and selected district-level statistics related to animal health officials.

Region	District reporting ASF	No. of wards	Staffing (involved in animal health issues)			Population at risk (pig)	Deaths reported due to ASF	Mortality rate (%)
			District office	Ward	Others (ward and village level)			
			(DVO/LO/LFO)	LFO	EO, AO, WEO, VEO			
Shinyanga	Kahama	20	1	9	NA	9,328	1820	19.5

Geita	Geita TC	13	2	6	NA	1,328	238	18.0
	Geita DC	37	5	11	NA	564	54	9.6
	Mbogwe	17	1	3	NA	1,567	388	24.7

Mwanza	Segerema	26	1	9	NA	2,591	444	17.1

Kagera	Ngara DC	22	1	12	NA	4,056	400	9.9
	Muleba DC	43	1	19	NA	4,233	105	2.5

Dodoma	Chamwino DC	36	5	17	4	6,120	1214	19.8
	Dodoma Jiji	41	6	30	3	3,161	141	4.5

Total		255	23	116	7	32,948	4,804	14.6

*Note*. DVOs and FGDs, March 2020; DVO = district veterinary officer; LO = livestock officer; LFO = livestock field officer; EO = extension officer; AO = agricultural officer; WEO = ward executive officer; VEO = village executive officer; TC = town council; DC = district council; NA = not available. All samples collected during the field exercise were confirmed using the partial amplification of major structural protein VP72 gene of ASF virus (ASFV). In total, 12 districts have reported outbreaks as at the time of field investigation.

**Table 2:** Expert opinion ranking of risk transmission pathways identified by the stakeholders for in-country and transboundary introduction and transmission of ASF virus.

Identified pathway	Ranking
Infected pigs movement (formal/informal) from affected areas within the country (Tanzania)	1
Contaminated pork product movement (formal/informal) from affected areas within the country (Tanzania)	2
Fomites (humans (service providers^*∗*^, other value chain actors and visitors), vehicles, and equipment)	3
Animal health practitioners, visitors and dog and cat movement within pig house, unsupervised slaughtering slabs, wild pigs/warthogs, and laboratory samples	3
Contaminated feeds (and drinking water) movement from affected areas within Tanzania	5
Infected live pigs imported (informal) through the international borders from areas known to be affected with ASF	6
Contaminated pork products (formal and informal) through the borders from areas known to be affected with ASF	7
Contaminated water/environmental materials from infected animals/products	8
Infected live pigs imported (informal) through the international borders from areas not known to be affected with ASF	9
Contaminated pork products (formal and informal) through the borders from areas not known to be affected with ASF	10
Infected live pigs imported (formal) through the international borders from areas known to be affected with ASF	10
Manure and beddings	12
Infected live pigs imported (formal) through the international borders from areas not known to be affected with ASF	12
Arthropods (flies, ticks, Stomoxys)	14
Laboratory personnel	15

*Note*. 1 = the riskiest and 15 = the least risky. Experts' opinions were provided based on selection of persons with significant contributions in the field of ASF research and diagnostics, field practice, teaching, and/or years of experience. All responses were based on independent empirical evaluations of ASF in farms. Full details of the reasoning behind the ranking are available in the Supplementary Table 4a. It should be understood that wild boars do not occur naturally in sub-Saharan Africa. The environment to pig cycle was described for a cycle identified in northern Europe where wild boars die of ASF and their carcasses contaminate the environment if not rapidly removed, helping to keep the infection going [13]. The ticks to pig cycles are related either to warthogs or to ticks that live in pigsties (as described in Malawi). Whether this affected the low ranking of arthropods and the relationship to wild pig was not evaluated. Perhaps, the ranking may differ in other territories. ^∗^Means veterinarians, para veterinarians, and input suppliers who are direct service providers to the farms.

**Table 3:** Expert opinion ranking of facilitators of ASF virus introduction and transmission to new premises for in-country and transboundary.

Facilitator	Ranking
Traders (whole pig)	1
Middlemen	2
Pig farmers	3
Transporters	4
Unauthorised animal health service providers, pork consumers, scavengers, and students	5
Traders (pork)	6
Veterinarians/para-veterinarians/livestock officers	7
Abattoir workers/butchers	8
Visitors	9
Agricultural officers/extension officers	10
Local government administrative officers	11
Border officials	12
Police and other control officers	13
Wild pig hunters	14
Feed manufacturers	15

*Note*. 1 = the most ranked and 15 = the least ranked. Experts' opinions were provided based on years of experience and empirical evaluation of ASF in farms. At subnational levels, sometimes, the agricultural officers, extension officers, and ward and village administrative staff serve as animal health officers and issue animal movement permits. Full details of the reasoning behind the ranking are available in the Supplementary Table 4b.

**Table 4:** Expert opinion ranking of susceptibility to hazard among the group of pigs in the farm in premises as identified by the stakeholders.

Pig group	Ranking
Shared adult boars	1
Pregnant and lactating sows (in pigs)	2
Nonpregnant sows	3
Nonshared adult boars	4
Growers	5
Weaners	6
Piglets^*∗*^	6
Porkers	8

*Note.* 1 = the most affected and 8 = the least affected. Stakeholders observed and provided anecdotal evidence that at the farm level, the subgroupings of pigs listed above have been affected to different degrees. It is hypothesized that different degrees of immunities in different pigs and the dose of infection may influence the degree of affection. Experts' opinions were provided based on years of experience and empirical evaluation of ASF in farms. ^*∗*^Piglets die typically due to starvation because of the death of sow. It should be noted that most dead piglets are not examined pathologically for causes of death. There was no significant difference between the rankings; hence, all subgroupings of pigs were classified as high in terms of susceptibility to the hazard. Full details of the reasoning behind the ranking are available in the Supplementary Table 4c.

## Data Availability

The data used in the publication are archived at the FAO, Tanzania, and the DVS, MoLF, Dodoma, Tanzania. Data can be provided on reasonable request to the Director of Veterinary Services, MoLF, Dodoma, Tanzania.

## Supplementary Materials

Supplementary Materials Supplementary Table 1: triage criteria for evaluation of the need for a rapid risk assessment. A threshold of 7 out of 14 was set (criteria 1–3: 1/2 + 3/6 + 3/6 = 7/14). The threshold value beyond which the triage score triggers an RRA varies depending on the capacity of the veterinary services to perform an RRA. For instance, if the veterinary services have the mandate and several staff members have the capacity and are available to perform an RRA, a lower threshold value will be set than when the veterinary services have few officers with the capacity and availability to conduct a RRA. Supplementary Table 2: farmer's self-rated questionnaire on biosecurity protocol in smallholder pig farms, Tanzania. Supplementary Table 3: expert opinion elicitation of risk transmission pathways for in-country and transboundary introduction and transmission of ASF virus. (i) Please rank the following risk transmission pathways for in-country and transboundary introduction and transmission of ASF virus (1 being the riskiest and the highest number being the least risky). Add any other, if known and rank too. Supplementary Table 4: expert opinion elicitation of facilitators of ASF introduction and transmission to new premises for in-country and transboundary introduction and transmission of ASF virus. (i) Please rank the following Facilitators of ASF introduction and transmission to new premises for in-country and transboundary introduction and transmission of ASF virus (1 being the riskiest and the highest number being the least risky). Add any other, if known and rank too. ^8 & 9^At subnational levels, sometimes, the agricultural officers, extension officers, and ward and village administrative staff serves as animal health officers and issue animal movement permits. Supplementary Table 5: expert opinion elicitation of hazard among the group of pigs in the farm premises. (i) Please rank the following hazard among the group of pigs in the farm in premises (1 being the most affected and the highest number being the least affected). Add any other category, if known and rank too. At the farm level, the subgroupings of pigs listed above were affected to different degrees. Based on your experience with ASF in farms, kindly rank them based on degree of affection providing reasons for your ranking. Supplementary Table 6: expert opinion elicitation of pathways and risk reduction measures to mitigate the burden of ASF in the farm premises. (1) Considering the table, kindly comment on the following: (a) are there pathways that should be added to the list? If yes, add it below. (b) Are the factors for consideration comprehensive enough? If not, can you modify appropriately tracking your responses? (c) Do you agree with the risk reduction measures? If not, what should be changed/added? ^*∗*^Service providers and other value chain actors include middlemen, transporters, pig farmers, traders (whole pig and pork), abattoir workers/butchers, LGA officials (vets and paraveterinarians, livestock officer, and agricultural extension officers), border officials, police, and control officers. Supplementary Table 7: identified risk pathways and basis for inclusion and scoring in the risk analysis. ^*∗*^Where an issue has been raised in the previous cell as a reason to justify the rank, it was not repeated in subsequent cells especially for common issues like importation and contamination. Note: *1* *=* the riskiest and 15 = the least risky. Experts' opinions were provided based on selection of persons with significant contributions in the field of ASF research and diagnostics, field practice, teaching and or years of experience. All responses were based on independent empirical evaluations of ASF in farms. Supplementary Table 8: identified facilitators and basis for inclusion and scoring in the risk analysis. Note: 1 = the most ranked and 15 = the least ranked. Experts' opinions were provided based on years of experience and empirical evaluation of ASF in farms. At subnational levels, sometimes, the agricultural officers, extension officers, ward and village administrative staff serve as animal health officers and issue animal movement permits. Supplementary Table 9: identified pig groups and basis for inclusion and scoring in the susceptibility analysis. Note: 1 = the most affected and 8 = the least affected. Stakeholders observed and provided anecdotal evidence that at the farm level, the subgroupings of pigs listed above have been affected to different degrees. It is hypothesized that different degrees of immunities in different pigs and the dose of infection may influence the degree of affection. Experts' opinions were provided based on years of experience and empirical evaluation of ASF in farms. ^*∗*^Piglets die typically due to starvation because of the death of sow. It should be noted that most dead piglets are not examined pathologically for causes of death. There was no significant difference between the rankings; hence, all subgroupings of pigs were classified as high in terms of susceptibility to the hazard. Supplementary Figure 1: rapid risk assessment (RRA) triaging process undertaken during the ASF mission in Tanzania, 2021 [5]. Supplementary Figure 2: classification of ASF virus based on the risk profile. Supplementary Figure 3: classification of ASF virus based on risk profile plausibility and likelihood. Supplementary Figure 4: schematic representation of interdistrict movement of pig and pig products. Partial representation of the map of Tanzania showing some of the districts in Kagera region and how interdistricts movement of pigs and pig products are operated. Supplementary Figure 5: schematic representation of intradistrict movement of pig and pig products. Supplementary Figure 6: sample of pig movements to and from the farms and towards the slaughter slabs and livestock markets.
