# Supplementary material for: Semiquantitative Risk Evaluation Reveals Drivers of African Swine Fever Virus Transmission in Smallholder Pig Farms and Gaps in Biosecurity, Tanzania
Source: Vet Med Int. 2024 May 13;2024:4929141. doi: 10.1155/2024/4929141 (PMC11105958; doi:10.1155/2024/4929141)
Supplement: Supplementary Materials — Supplementary Table 1: triage criteria for evaluation of the need for a rapid risk assessment. A threshold of 7 out of 14 was set (criteria 1–3: 1/2 + 3/6 + 3/6 = 7/14). The threshold value beyond which the triage score triggers an RRA varies depending on the capacity of the veterinary services to perform an RRA. For instance, if the veterinary services have the mandate and several staff members have the capacity and are available to perform an RRA, a lower threshold value will be set than when the veterinary services have few officers with the capacity and availability to conduct a RRA. Supplementary Table 2: farmer's self-rated questionnaire on biosecurity protocol in smallholder pig farms, Tanzania. Supplementary Table 3: expert opinion elicitation of risk transmission pathways for in-country and transboundary introduction and transmission of ASF virus. (i) Please rank the following risk transmission pathways for in-country and transboundary introduction and transmission of ASF virus (1 being the riskiest and the highest number being the least risky). Add any other, if known and rank too. Supplementary Table 4: expert opinion elicitation of facilitators of ASF introduction and transmission to new premises for in-country and transboundary introduction and transmission of ASF virus. (i) Please rank the following Facilitators of ASF introduction and transmission to new premises for in-country and transboundary introduction and transmission of ASF virus (1 being the riskiest and the highest number being the least risky). Add any other, if known and rank too. 8 & 9At subnational levels, sometimes, the agricultural officers, extension officers, and ward and village administrative staff serves as animal health officers and issue animal movement permits. Supplementary Table 5: expert opinion elicitation of hazard among the group of pigs in the farm premises. (i) Please rank the following hazard among the group of pigs in the farm in premises (1 being the most affected an [file 4929141.f1.docx]

**Supplementary materials**

**Supplementary Tab****le 1. Triage criteria for evaluation of the need for a rapid risk assessment**

| *Criteria* | *Score (Yes = 1; No = 0)* | | |
| --- | --- | --- | --- |
|  | *Yes* | *No* | *Unknown* |
| ***1. Credibility of data sources*** | | | |
| *Has the health event been reported by multiple independent and reliable unofficial sources (e.g., the media, ProMED,* field sources, twitter)?* | *X* |  |  |
| *Has the health event been reported or confirmed by an official source?* | *X* |  |  |
| ***Subtotal score*** | ***2*** | | |
| ***2. Severity of the disease and its consequences*** | | | |
| *Has the international spread of the disease (via live animals or their products, vectors or fomites) been proved?* | *X* |  |  |
| *Has the disease been shown to have a significant impact on the health of domestic animals in multiple countries?* | *X* |  |  |
| *Is it a zoonotic disease associated with severe consequences for public health?* |  | *X* |  |
| *Has the disease been shown to have a significant impact on animal production and/or trade with possible detrimental economic consequences for the affected country or countries?* | *X* |  |  |
| *Has the disease been shown to have a significant impact on the health of wildlife or on the environment, including biodiversity, in one or more countries?* | *X* |  |  |
| *Has the causative agent of the disease developed resistance to treatments, thereby posing a significant danger to public and/or animal health?* |  | *X* |  |
| ***Subtotal score*** | ***4*** | | |
| ***3. Relevance of the health event*** | | | |
| *Is the observed health event possibly linked to the evolution or change of an existing disease agent?* |  | *X* |  |
| *Is the observed health event related to the spread of a known disease to a new geographic area, species or population?* | *X* |  |  |
| *Is the observed health event related to a known disease that is occurring with increased incidence or morbidity in the host population(s)?* | *X* |  |  |
| *Is the observed health event caused by an unknown or previously unrecognized disease agent?* |  | *X* |  |
| *Is the observed health event affecting vulnerable groups of the population, such as infants or elderly people, who are likely to be disproportionately affected?* |  | *X* |  |
| *Is the observed health event attracting a high actual or potential level of media interest or public concern?* | *X* |  |  |
| ***Subtotal score*** | ***3*** | | |
| ***Total*** | ***9/14*** | | |

*A threshold of 7 out of 14 was set (Criteria 1-3: 1/2 + 3/6 + 3/6 = 7/14).* *The threshold value beyond which the triage score triggers an RRA varies depending on the capacity of the veterinary services to perform an RRA. For instance, if the veterinary services have the mandate and several staff members have the capacity and are available to perform an RRA, a lower threshold value will be set than when the veterinary services have few officers with the capacity and availability to conduct a RRA.*

Supplementary Table 2. Farmer’s self-rated questionnaire on biosecurity protocol in smallholder pig farms, Tanzania

| **Biosecurity Variable** | Response |
| --- | --- |
|  | YES(Y)/NO (N) |
| 1: Restricted access to all visitors |  |
| 2: Fence around premises |  |
| 3: Gate at entrance |  |
| 4: Change solution in foot pans regularly |  |
| 5: Record keeping |  |
| 6: Food and water control |  |
| 7: Routine (regular) cleaning |  |
| 8: Safe disposal of feces and dead pigs (protected away from other animals and insects) |  |
| 9: Provision of quarantine of newly purchased and other introduced pigs for at least 10 days |  |
| 10: Sufficient space for each pig (no overcrowding) |  |
| 11: Usage of disinfectant after cleaning |  |
| 12: Assess health status of pigs coming in |  |
| 13: Do not mix different ages |  |
| 14: Do not mix different species |  |
| 15: All-in-all-out production |  |
| 16: Going from young to older pigs |  |
| 17: Change clothes when going in/out |  |
| 18: Separate sick pigs |  |
| 19: Consult with a veterinary in case of sick pigs |  |
| 20: Change rubber boots/slippers |  |
| 21: Wash/disinfect of other equipment and tools |  |
| 22: Downtime>=2 |  |
| 23: Pest control (rodents and insects) |  |
| 24: Auditing: incentives, education, adherence (encourage assistants to adhere to biosecurity) |  |
| 25: Sampling before and after transportation |  |

***Supplementary Table 3. Expert Opinion Elicitation of Risk transmission pathways for in-country and transboundary introduction and transmission of ASF virus.***

- Please rank the following ***Risk transmission pathways*** for in-country and transboundary introduction and transmission of ASF virus. (***1 being the riskiest and the highest number being the least risky***). Add any other, if known and rank too.

|  | **Identified pathway** | **Rank** | **Reason** |
| --- | --- | --- | --- |
|  | Infected live pigs ***imported*** (**formal**) through the international borders from areas **not known** to be affected with ASF |  |  |
|  | Infected live pigs ***imported*** (**informal**) through the international borders from areas **not known** to be affected with ASF |  |  |
|  | Infected live pigs ***imported*** (**formal**) through the international borders from areas **known** to be affected with ASF |  |  |
|  | Infected live pigs ***imported*** (**informal**) through the international borders from areas **known** to be affected with ASF |  |  |
|  | ***Contaminated pork products*** (formal and informal) through the borders from areas **not known** to be affected with ASF |  |  |
|  | ***Contaminated pork products*** (formal and informal) through the borders from areas **known** to be affected with ASF |  |  |
|  | Infected ***pigs movement*** (formal/informal) from affected areas within the country (Tanzania) |  |  |
|  | ***Contaminated pork product movement*** (formal/informal) from affected areas within the country (Tanzania) |  |  |
|  | ***Contaminated feeds*** (and drinking water) movement from affected areas within Tanzania |  |  |
|  | ***Fomites*** (humans – (service providers*, other value chain actors and visitors), vehicles and equipment) |  |  |
|  | ***Contaminated water/ environmental materials*** from infected animals/products |  |  |
|  | ***Manure and beddings*** |  |  |
|  | ***Arthropods*** (flies, ticks, Stomoxys) |  |  |
|  | ***Others:*** *(please list)* |  |  |
|  |  |  |  |

***Supplementary Table 4. Expert Opinion Elicitation of Facilitators of ASF introduction and transmission to new premises for in-country and transboundary introduction and transmission of ASF virus.***

- Please rank the following ***Facilitators of ASF introduction and transmission to new* premises** for in-country and transboundary introduction and transmission of ASF virus. (***1 being the riskiest and the highest number being the least risky***). Add any other, if known and rank too.

|  | **Facilitators** | **Rank** | **Reason** |
| --- | --- | --- | --- |
|  | Middlemen |  |  |
|  | Transporters |  |  |
|  | Pig farmers |  |  |
|  | Traders (whole pig) |  |  |
|  | Traders (pork) |  |  |
|  | Abattoir workers/ butchers |  |  |
|  | Veterinarians/ para-veterinarians/ Livestock officers |  |  |
|  | Agricultural officers /extension officers |  |  |
|  | Local government administrative officers |  |  |
|  | Border officials |  |  |
|  | Police and other control officers |  |  |
|  | Visitors |  |  |
|  |  |  |  |

*^8 & 9^At subnational levels, sometimes, the agricultural officers, extension officers, ward and village administrative staff serves as animal health officers and issue animal movement permits.*

***Supplementary Table 5. Expert Opinion Elicitation of Hazard among group of pigs in the farm premises.***

- Please rank the following ***Hazard among group of pigs in the farm in premises***. (***1 being the most affected and the highest number being the least affected***). Add any other category, if known and rank too.

|  | **Pig group** | **Rank** | **Reason** |
| --- | --- | --- | --- |
|  | Pregnant sows (in-pigs) |  |  |
|  | Non-pregnant sows |  |  |
|  | Shared adult boars |  |  |
|  | Non-shared adult boars |  |  |
|  | Porkers |  |  |
|  | Growers |  |  |
|  | Weaners |  |  |
|  | Piglets |  |  |

*At farm-level, the subgroupings of pigs listed above were affected to different degrees. Based on your experience with ASF in farms, kindly rank them based on degree of affection providing reasons for your ranking.*

***Supplementary Table 6. Expert Opinion Elicitation of Pathways and Risk reduction measures to mitigate the burden of ASF in the farm premises.***

1. **Considering the table below, kindly comment on the following:**
2. Are there **pathways** that should be added to the list? If yes, add it below.
3. Are the **factors for consideration** comprehensive enough? If not, can you modify appropriately tracking your responses?
4. Do you agree with the risk reduction measures? If not, what should be changed/added?

| **Pathway** | **Factors for consideration** | **Risk reduction measures** |
| --- | --- | --- |
| Infected live pigs ***imported*** (**formal**) through the international borders from areas **not known** to be affected with ASF | - live pig movement patterns  - Sensitivity of surveillance & vigilance in exporting area  - Timeliness of outbreak reporting from exporting areas  - Adherence to import regulations | - Improved disease intelligence and dialogue with neighbouring countries.  - Strict enforcement of import regulations  - Improvement of skill and competence of border officials  - Improved understanding of the risks and consequences through stakeholder education  - Stakeholders’ awareness and training |
| Infected live pigs ***imported*** (**informal**) through the international borders from areas **not known** to be affected with ASF | - live pig movement patterns  - Sensitivity of surveillance & vigilance in exporting area  - Timeliness of outbreak reporting from exporting areas  - Adherence to import regulations | - Improved disease intelligence and dialogue with neighbouring countries.  - Strict enforcement of import regulations  - Improvement of skill and competence of border officials  - Improved understanding of the risks and consequences through stakeholder education  - Stakeholders’ awareness and training |
| Infected live pigs ***imported*** (**formal**) through the international borders from areas **known** to be affected with ASF | - live pig movement patterns  - Sensitivity of surveillance & vigilance in exporting area  - Timeliness of outbreak reporting from exporting areas  - Adherence to import regulations | - Improved disease intelligence and dialogue with neighbouring countries.  - Strict enforcement of import regulations  - Improvement of skill and competence of border officials  - Improved understanding of the risks and consequences through stakeholder education  - Stakeholders’ awareness and training |
| Infected live pigs ***imported*** (**informal**) through the international borders from areas **known** to be affected with ASF | - live pig movement patterns  - Sensitivity of surveillance & vigilance in exporting area  - Timeliness of outbreak reporting from exporting areas  - Adherence to import regulations | - Improved disease intelligence and dialogue with neighbouring countries.  - Strict enforcement of import regulations  - Improvement of skill and competence of border officials  - Improved understanding of the risks and consequences through stakeholder education  - Stakeholders’ awareness and training |
| ***Contaminated pork products*** (formal and informal) through the borders from areas **not known** to be affected with ASF | - identified pork products’ movement patterns  - Sensitivity of surveillance and vigilance in exporting area  - Timeliness of outbreak reporting from exporting areas  - Adherence to import regulations | - Improved awareness and sensitization of all actors along the pig value chain regarding the risks and consequences  - Improved disease intelligence and dialogue with neighbouring countries  - Strict enforcement of import regulations  - Improvement of skill and competence of border officials |
| ***Contaminated pork products*** (formal and informal) through the borders from areas **known** to be affected with ASF | - identified pork products’ movement patterns  - Sensitivity of surveillance and vigilance in exporting area  - Timeliness of outbreak reporting from exporting areas  - Adherence to import regulations | - Improved awareness and sensitization of all actors along the pig value chain regarding the risks and consequences  - Improved disease intelligence and dialogue with neighbouring countries  - Strict enforcement of import regulations  - Improvement of skill and competence of border officials |
| Infected ***pigs movement*** (formal/informal) from affected areas within the country (Tanzania) | - pig husbandry practices  - live pig movement patterns  - Pig movement controls  - pig and pig products value chain  - Sensitivity of surveillance  - Timeliness of reporting | - Advocate Good Husbandry Practices (including professional guidance and testing during purchases, selling and other related movements in and out of the farm etc.)  - Intensify targeted surveillance and prompt reporting of outbreaks  - Strict enforcement of movement controls  - Improved awareness and sensitization of all actors along the pig value chain regarding the risks and consequences  - Behavioural change communication along the value chain |
| ***Contaminated pork product movement*** (formal/informal) from affected areas within the country (Tanzania) | - pig husbandry practices  - pork product movement patterns  - Pork product movement controls  - Sensitivity of surveillance  - Timeliness of reporting | - Improved awareness and sensitization of all actors along the pig value chain regarding the risks and consequences  - Strict enforcement of movement controls  - Intensify targeted surveillance and prompt reporting of outbreaks  - Behavioural change communication along the value chain |
| ***Contaminated feeds*** (and water) movement from affected areas within the Tanzania | - feeds movement patterns  - downstream flow of contaminated waters  - feeds movement controls  - Sensitivity of surveillance  - Timeliness of reporting | - Strict enforcement of feed controls  - Improved awareness and sensitization of all actors along the pig value chain regarding the risks and consequences  - Biosecurity improvement (e.g. minimize exposure of contaminated feeds to pigs)  - Improved and safe water sources  - Behavioural change communication along the value chain |
| ***Fomites*** (humans – (service providers*, other value chain actors and visitors), vehicles and equipment) | - Adherence to good pig husbandry practices and movement patterns  - provisions of biosecurity measure at farm level  - Services providers should ensure biosecurity and reduction of risk of inadvertent spread from farm to farm. | - Advocate Good Husbandry Practices  - Improve biosecurity practices (e.g. require cleaning and disinfection of personnel, contaminated vehicles)  - Surveillance intensification  - Biosecurity improvements  - Improved awareness and sensitization of all actors along the pig value chain regarding the risks and consequences  - Behavioural change communication along the value chain |
| ***Contaminated water/ environmental*** from infected animals/products | - Pig husbandry practices and movement patterns  - Burial and disposal practices for carcasses  - unregulated slaughter (e.g. own farm slaughter)  - scavenger discouragement around farms | - Advocate Good Husbandry Practices  -Advocate for establishment of slaughter slabs/facilities where good hygiene practices is implemented & monitored  - Improved awareness and sensitization of all actors along the pig value chain regarding the risks and consequences  - Awareness on the Guidelines for Carcass disposal document from FAO. |
| ***Manure and beddings*** | - Pig husbandry practices and movement patterns  - Disposal practices for manure and beddings | - Advocate Good Husbandry Practices  - Advocate for creation of disposal pits in farms  - Advocate for proper disposal of visceral content post-slaughtering  - Awareness on the Guidelines for Carcass disposal document from FAO. |
| ***Arthropods*** (flies, ticks, Stomoxys) | - Pig husbandry practices and movement patterns  - Fencing around pig premises  - wild boars/warthogs discouragement around farms | - Advocate Good Husbandry Practices  -Advocate for fencing of farms  - Improved awareness and sensitization of all actors along the pig value chain regarding the risks and consequences  - Awareness on the Guidelines for Carcass disposal document from FAO. |
| ***Others:*** *(please list)* |  |  |

**service providers and other value chain actors include middlemen, transporters, pig farmers, traders (whole pig, pork), abattoir workers/butchers, LGA officials (vets and para-veterinarians, livestock officer, agricultural extension officers), border officials, police and control officers among others.*

**Supplementary Table 7. Identified risk pathways and basis for inclusion and scoring in the risk analysis.**

| **s/no.** | **Identified pathway** | **Reason*** |
| --- | --- | --- |
|  | Infected live pigs ***imported*** (**informal**) through the international borders from areas **not known** to be affected with ASF | Although, the importation of pigs was banned in Tanzania, the informal pig movements across borders are highly unregulated and easily expose naïve pig populations to infection, e.g Tanzania-Zambia routes.  Timeliness of outbreak reporting from exporting areas and adherence to import regulations, poor disease intelligence and dialogue with neighbouring countries affects detections.  This is high in the border districts due to cultural/trade similarities across borders.  Most of Tanzania’s neighbours are known to be infected with ASF and there have been outbreaks linked to outbreaks in Malawi and possibly Burundi. |
|  | ***Contaminated pork products*** (formal and informal) through the borders from areas **not known** to be affected with ASF | Few trade pathways follow this route especially the formal route. There are some chances of the contaminated pork going through the informal route albeit it being also low.  During outbreak, domestic pigs that died from ASF may be slaughtered to minimize economic loss. In that case, the contaminated pork products play a crucial role in the transmission of ASF from an infected area to other localities.  Poorly identified pork products’ movement patterns and non-adherence to import regulations hampers progress.  Only effective in-country surveillance can detect ASF virus.  Sometimes, transportation of pork (pig meat) from one area to another is done by mixing such with crops like banana and maize in order to avoid being caught by veterinary officers at the checkpoints during quarantine period.  Mislabelling of infected products may be practised.  Infected pork is the second most frequent cause of outbreaks in pigs, usually through scavenging or swill feeding; carcasses and swill ingredients usually local. |
|  | Infected live pigs ***imported*** (**formal**) through the international borders from areas **known** to be affected with ASF | Low chances of clinically infected pigs passing through controlled borders. The major chance of going through the borders is if the pig is in a subclinical infection stage while going through the border.  Poor pig husbandry practices, uncontrolled pig movement and inadequate enforcements of movements (quarantine). Poor field disease diagnosis and management.  Inadequate level of staffing and sometimes overrule of empirical decisions by high rank officials limit success. |
|  | Infected live pigs ***imported*** (**informal**) through the international borders from areas **known** to be affected with ASF | This is a common practice across borders with the same communities that have a lot to share in common. This has been experienced between many countries in the eastern Africa region i.e. Tanzania-Malawi, Tanzania-Zambia, Tanzania-DRC, Kenya-Uganda, Tanzania-Uganda, Uganda-DRC, Tanzania-Burundi, Burundi Rwanda, Uganda-Rwanda.  Poor pig husbandry practices, uncontrolled pig movement and inadequate enforcements of movements (quarantine). Poor field disease diagnosis and management. |
|  | ***Contaminated pork products*** (formal and informal) through the borders from areas **known** to be affected with ASF | This is a common occurrence. Occurs when sick and infected pigs are slaughtered to avoid losses by farmers during quarantines and is usually seen in borders with same communities across the countries.  Illegal trade, and the abundance of meat promotes donation and sharing both in surrounding localities but often over exceptionally long distances, creating new loci of outbreaks.  The sharing of meat during ceremonies is also common (funerals, weddings, etc.). Piece of meat brought at home, if contaminated, can create new ASF outbreaks when pigs consume leftovers. |
|  | ***Contaminated pork product movement*** (formal/informal) from affected areas within the country (Tanzania) | Occurs in many locations and there will be a high chance of transmitting the infection. |
|  | ***Contaminated feeds*** (and drinking water) movement from affected areas within Tanzania | When an outbreak occurs in a particular location causing massive death of pigs, the unused feeds are sent or sold out to pig owners in other areas.  Not a very common pathway as feeds are rarely shared between locations during peace times. However, swill, which is a common source of infection is shared.  Inadequate knowledge on feed quality and regimen, no specific feeds for pigs, and usage of effluent water in piggeries.  The risk of transmission from these sources is comparatively low. The highest risk from contaminated feed obtained locally is to accept leftover feed from a farmer whose pigs have died of ASF.  The risk of contamination through food is common and high. It can occur through carrier bags, trucks which transport both pigs and feed or raw materials and which are not sufficiently cleaned and disinfected, etc. |
|  | ***Contaminated water/ environmental materials*** from infected animals/products | Occurs but with very minimal chances of transmission. Not a common pathway in the region. Water is often cited as a possible source but this is only likely if the source is of low volume, stagnant and heavily contaminated.  Slaughter facilities within the pig farm premises, collection of animals from different sources, and discharge of potentially contaminated effluents into streams.  In parts of Tanzania, most outbreaks occur during rainy seasons for the following reasons; effluents from slaughter slabs may flow into streams, and pig farmers may dispose dead pigs in flowing streams with subsequent downstream flow of infections.  These reasons need further investigations. |
|  | Infected ***pigs movement*** (formal/informal) from affected areas within the country (Tanzania) | This occurs in many locations including night transportation, hence the risk of transmission along the route. This is so especially when sub clinical infected pigs are transported.  Domestic pig cycle is the main mode of transmission.  Vehicles meant for transport are shared between transporting pigs and for many other purposes.  Infected pigs are the most potent source of infection and movement within Tanzania has been identified as a cause of outbreaks more frequently than cross-border movement.  Poor stakeholder engagements. |
|  | ***Arthropods*** (flies, ticks, Stomoxys) | Very low chance of spreading the disease as it is not a very common route.  Due to scarcity of land, the owners of pigs keep them in limited area as clusters of farms, so, an infection of ASF in one farm may be introduced to other farms by arthropods and insects, although the role of insects are yet to be proven. It will be a good research to engage in.  Soft ticks *Ornithodorus moubata* is the main vector which play role in transmission of the disease |
|  | ***Manure and beddings*** | A very common occurrence and can lead to fast transmission of the virus as manure is used for crop production during rainy season as stated above and may inadvertently transport the virus from one point to another.  Manure, especially in warm sunny climates, is not considered a high risk as virus shedding in manure is sporadic at best and occurs if the faeces contain blood; accepting used bedding from a farmer whose pigs had died of ASF could pose a risk. |
|  | ***Fomites*** (humans – (service providers*, other value chain actors and visitors), vehicles and equipment) | This occurs but is mainly transmitted by animal health service providers and vehicles that are used to transport infected pigs.  Inadequate knowledge on disease transmission.  The most frequently observed factors come from human intervention. The greatest risk factor is human misconduct by all actors in the pork value chain. These include poor farming practices, non-compliance with biosecurity and unsuitable or even deviant social behavior.  Based on experience, during an outbreak and death of pigs, farmers inform neighbours, who often come to commiserate or empathize, and may inadvertently carry infection back to their farms.  The anthropogenic factor is important in the transmission of ASF in our region, mainly visitors, service providers and traders. The equipment sharing between farms also play a not negligible role in the transmission of ASF between farms.  Apart from the biological vector tick (*Ornithodoros*) that lives in warthog burrows and sometimes pig shelters, the only arthropod of very many examined (including hard ticks, house flies and blow flies) that has experimentally been shown able to transmit the virus is *Stomoxys*, but to date we do not know of any outbreak caused by these small flies. |
|  | Infected live pigs ***imported*** (**formal**) through the international borders from areas **not known** to be affected with ASF | Very low chances of clinically infected pigs passing through controlled borders. The major chance of going through the borders when subclinical infected pigs go through the border after going through the non-infected areas.  Compliance with international regulations in that matter, Surveillance, vigilance, issuance of a health certificate, quarantine, etc. is necessary. |
|  | Animal health practitioners, visitors and dog and cat movement within pig house, unsupervised slaughtering slabs, wild pigs/warthogs and laboratory samples | Most of dogs and other scavengers in rural area are not confined and move from one area to another in search of food; they sometimes pick contaminated pig products back into pig houses and may be a source of multi-farm transmission.  Poor hygienic measures, poor sanitation, lack of pig and meat inspection.  Around the wildlife parks, conservations and reserve areas, the sylvatic mode of ASF transmission may be major risk and possibility in the interfaces. |
|  | Laboratory personnel | If not properly decontaminate/wear the PPEs, subsequent visit to other farms may introduce infection. |

*Where an issue has been raised in the previous cell as a reason to justify the rank, it was not repeated in subsequent cells especially for common issues like importation and contamination. Note: *1 = the riskiest and 15 = the least risky*. *Experts’ opinions were provided based on selection of persons with significant contributions in the field of ASF research and diagnostics, field practice, teaching and or years of experience. All responses were based on independent empirical evaluations of ASF in farms.*

**Supplementary Table 8. Identified facilitators and basis for inclusion and scoring in the risk analysis**

|  | **Facilitator** | **Reason** |
| --- | --- | --- |
|  | Traders (whole pig) | They meet most of the stakeholders in the value chain. Inadequate knowledge of the disease and its mode of transmission, movement from one farm to the next. |
|  | Middlemen | Inadequate knowledge of the disease and its mode of transmission, movement from one farm to the next.  They are the second in terms of getting in contact with pig value chain actors, and are involved in smuggling.  They buy pigs from the ASF-infected area and send to ASF-free area, or move around with them. pig keepers are forced to sell their pigs during outbreaks to reduce or avoid losses |
|  | Pig farmers | They usually move from one farm to another and markets and can spread the infection fast.  Inadequate knowledge of the disease and its mode of transmission, movement from one farm to the next. |
|  | Transporters | They traverse long distance and can spread the infection everywhere.  Inadequate knowledge of the disease and its mode of transmission, movement from one farm to the next |
|  | Unauthorised animal health service providers, pork consumers, scavengers and students | Tanzania has few animal health officers, so pig keepers used to these people (VISHOKA) who do not follow key principles of animal health causing spread of the disease to other areas because these are the first person to be contacted without knowing which disease certain pig is suffering from. Anecdotal evidence and field reports confirmed that these individuals treat all cases with antibiotics first and only when these fails do they advise farmers to consult with veterinarians. |
|  | Traders (pork) | They spread the disease especially when they engage in informal trade where infected pigs are sold in clandestine manner.  Inadequate knowledge of the disease and its mode of transmission, movement from one farm to the next* |
|  | Veterinarians/ para-veterinarians/ Livestock officers | They usually move from farm to farm to treat and to market products, and could spread disease in case of poor biosecurity.  Misdiagnoses and wrong differentials of diseases during outbreaks.  Professionals also use outbreak situation to teach students and junior professionals and this may inadvertently spread infection.  Have high level of knowledge on biosecurity measures, specific to the different pig producing sectors like cleaning and disinfection of farms, transport vehicles, and improved husbandry practices and production systems, but the application may be wanting. |
|  | Abattoir workers/ butchers | Can inadvertently transmit ASF virus when they get in contact with infected meat/products and then move to ASF-free pig farms/homesteads.  Inadequate knowledge of the disease and its mode of transmission, movement from one farm to the next  These workers buy pigs for slaughter from different villages, wards using the same vehicle to collect pigs from different sources without observing biosecurity. It is believe (not confirmed) that unscrupulous workers also spread ASF purposely in order to get cheap pigs to buy or to have higher bargaining power. |
|  | Visitors | They can transmit from one farm to another when they do not adhere to the laid down biosecurity measures.  Inadequate knowledge of the disease and its mode of transmission. |
|  | Agricultural officers /extension officers | They move from farm to farm and could spread the disease in case they get in contact with infected premises / animals  Inadequate knowledge of the disease and its mode of transmission |
|  | Local government administrative officers | Though minimal, can spread the disease when they get in touch with infected materials either in the farms or local authority markets  Inadequate knowledge of the disease and its mode of transmission, yet they may issue movement permits at local levels.  Sometimes visit farmers to observe losses for accountability. |
|  | Border officials | Very minimal chances when they get in touch with especially sub clinically infected pigs along the formal trade routes across the borders.  Inadequate knowledge of the disease and its mode of transmission, yet they may issue movement permits at the borders or local levels |
|  | Police and other control officers | Very minimal chances except when they get in touch with especially sub-clinically infected pigs along the formal trade routes across the borders.  May sometimes collect bribes and allow traders and farmers to move with pigs during quarantine and movement restrictions. |
|  | Wild pig hunters | Thought to be rare, because game is not a popular meat in Tanzania. However, they may inadvertently transmit infection especially at the interface areas. |
|  | Feed manufacturers | These may transmit infection during supplies of feed from farm to farms. It is also possible when feed bags from infected farms are brought to the feed mill. |

Note: *1 = the most ranked and 15 = the least ranked*. Experts’ opinions were provided based on years of experience and empirical evaluation of ASF in farms. *At subnational levels, sometimes, the agricultural officers, extension officers, ward and village administrative staff serve as animal health officers and issue animal movement permits.*

**Supplementary Table 9. Identified pig groups and basis for inclusion and scoring in the susceptibility analysis**

|  | **Pig group** | **Reason** |
| --- | --- | --- |
|  | Pregnant and lactating sows (in-pigs) | Least in movement but this disease kills any group of pigs.  Frequent care by farmer, much more sensitive animals, contact between pregnant sows of the same pen, increased probability of contamination. |
|  | Non-pregnant sows | Do not move much as for the younger age categories  Promiscuity and frequent contact between sows, with increased probability of contamination. |
|  | Shared adult boars | Can transmit ASF from infected premise to many farms because of movement from farm to farm.  Boar with visually good appearance are borrowed by several pig farmers for mating purpose and may get infected with ASF virus inadvertently and share same to other farms. |
|  | Porkers | Highest chances of being sold and moved from one farm to another farm and market could lead to transmission of ASF.  Large concentration of animals, and frequent contact between pigs, which increases probability of contamination. Access by pig dealers to select or buy animals for the market. |
|  | Non-shared adult boars | Movement from farm to farm limited |
|  | Weaners | Have high chance of movement between farms and markets  Large concentration of animals grouped after selection from several compartments. Frequent care by farmer, contact between pigs, with fights and injuries, which increases probability of contamination. |
|  | Growers | Have medium chance of movement between farms and markets  Large concentration of animals, Promiscuity, and frequent contact between pigs, with fights and injuries, with increased probability of contamination. |
|  | Piglets* | Low chances of being moved between farms and markets  Usually isolated with the suckling sow in an individual pen, the probability of contamination is low, except by the intervention of the farmer. |

Note: ***1 = the most affected and 8 = the least affected***. *Stakeholders observed and provided anecdotal evidence that at farm-level, the subgroupings of pigs listed above have been affected to different degrees. It is hypothesized that different degrees of immunities in different pigs and the dose of infection may influence the degree of affection. Experts’ opinions were provided based on years of experience and empirical evaluation of ASF in farms. *Piglets die typically due to starvation because of the death of sow. It should be noted that most dead piglets are not examined pathologically for causes of death. There was no significant difference between the rankings; hence, all subgroupings of pigs were classified as high in terms of susceptibility to the hazard.*


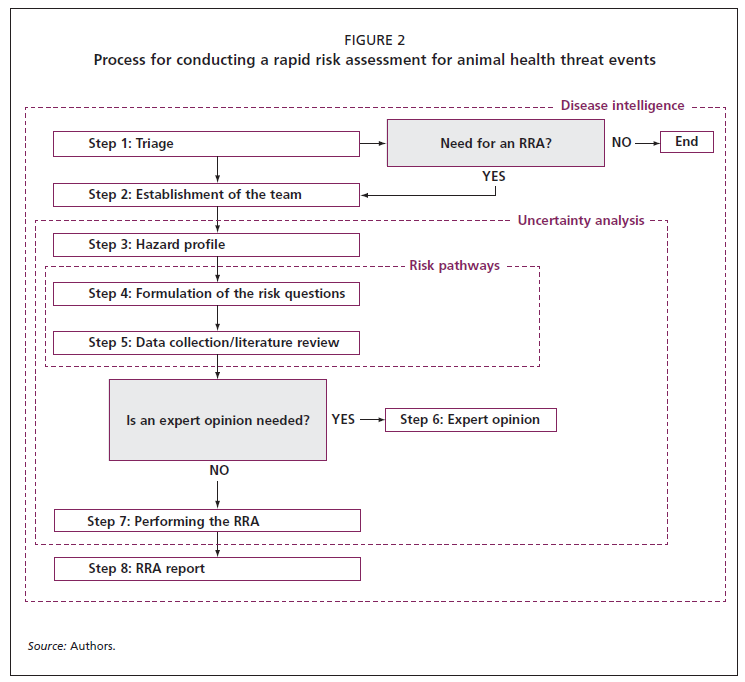


Supplementary Figure 1: Rapid risk assessment (RRA) triaging process undertaken during the ASF mission in Tanzania, 2021, [5].

- **Classification of ASF virus based on risk profile**

|  | **Classification of ASF virus based on risk profile** | **Strongly disagreed** | | **Disagreed** | | **Neutral** | | **Agreed** | | **Strongly agreed** | |
| --- | --- | --- | --- | --- | --- | --- | --- | --- | --- | --- | --- |
| 1 | *Based on discussion with farmers and other stakeholders in Tanzania, ASF virus and the disease was classified as disease with* ***very high risk*** *among the smallholder farms (see schematic below), first****, do you agree with this classification?*** *If yes, using your knowledge of ASF in Tanzania and elsewhere,* ***how much do you agree with this on a scale of 1-10? If no, where will you classify ASF****? Your note is welcomed below.* | 1 | 2 | 3 | 4 | 5 | 6 | 7 | 8 | 9 | 10 |

| **Yes** |  | **No** |  |
| --- | --- | --- | --- |


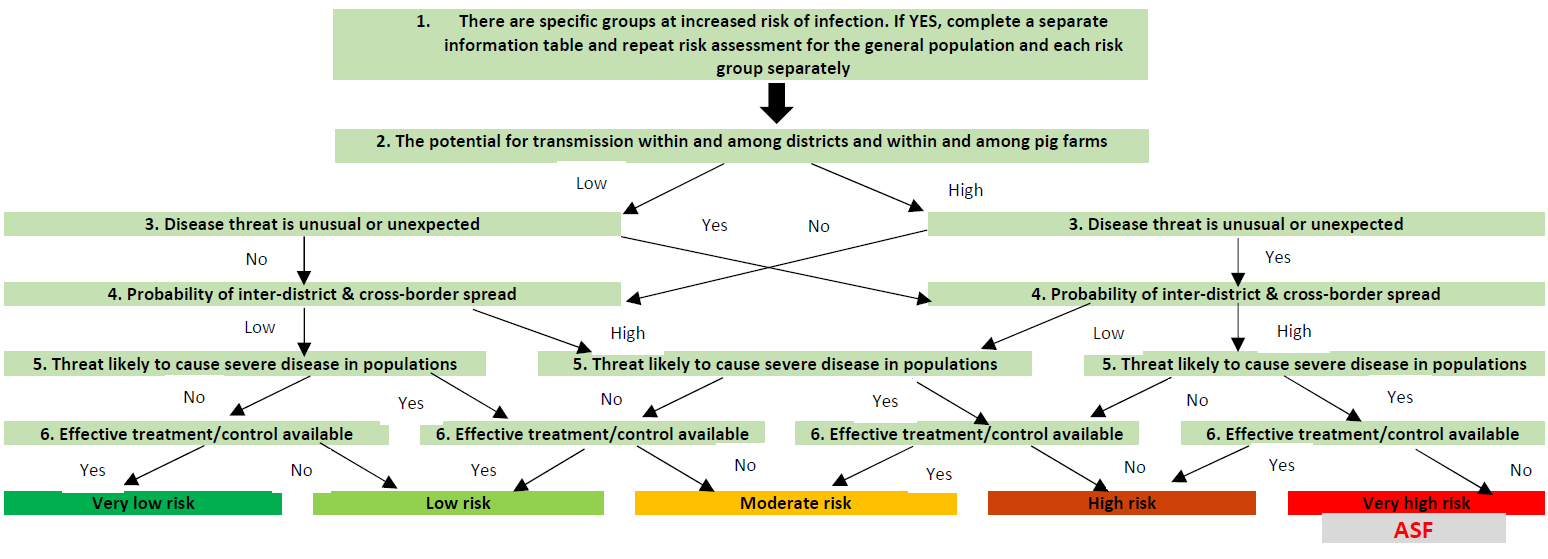


***Supplementary Figure 2. Classification of ASF virus based on risk profile.***

- **Classification of ASF virus based on risk profile.**

|  | **Classification of ASF virus introduction based on plausibility and likelihood** | **Strongly disagreed** | | **Disagreed** | | **Neutral** | | **Agreed** | | **Strongly agreed** | |
| --- | --- | --- | --- | --- | --- | --- | --- | --- | --- | --- | --- |
| 1 | *Based on discussion with farmers and other stakeholders in Tanzania, ASF virus introduction and transmission pathways among the smallholder farms was proposed as below (see schematic below), on a scale of 1-10, how much do you agree with this? Your comment is welcomed.* | 1 | 2 | 3 | 4 | 5 | 6 | 7 | 8 | 9 | 10 |

***Supplementary Figure 3. Classification of ASF virus based on risk profile plausibility and likelihood.***


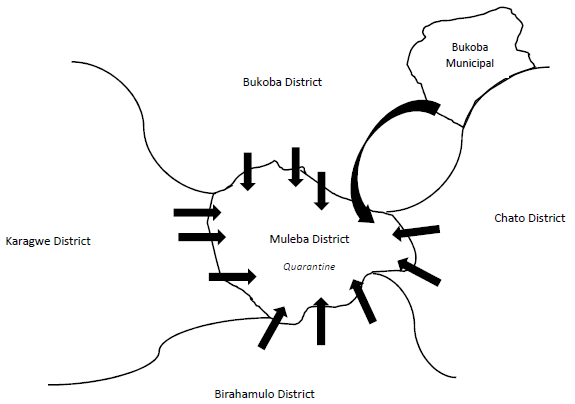


Supplementary Figure 4: **Schematic representation of inter-district movement of pig and pig products**

*Partial representation of the map of Tanzania showing some of the Districts in Kagera Region and how inter-districts movement of pigs and pig products are operated.*

*
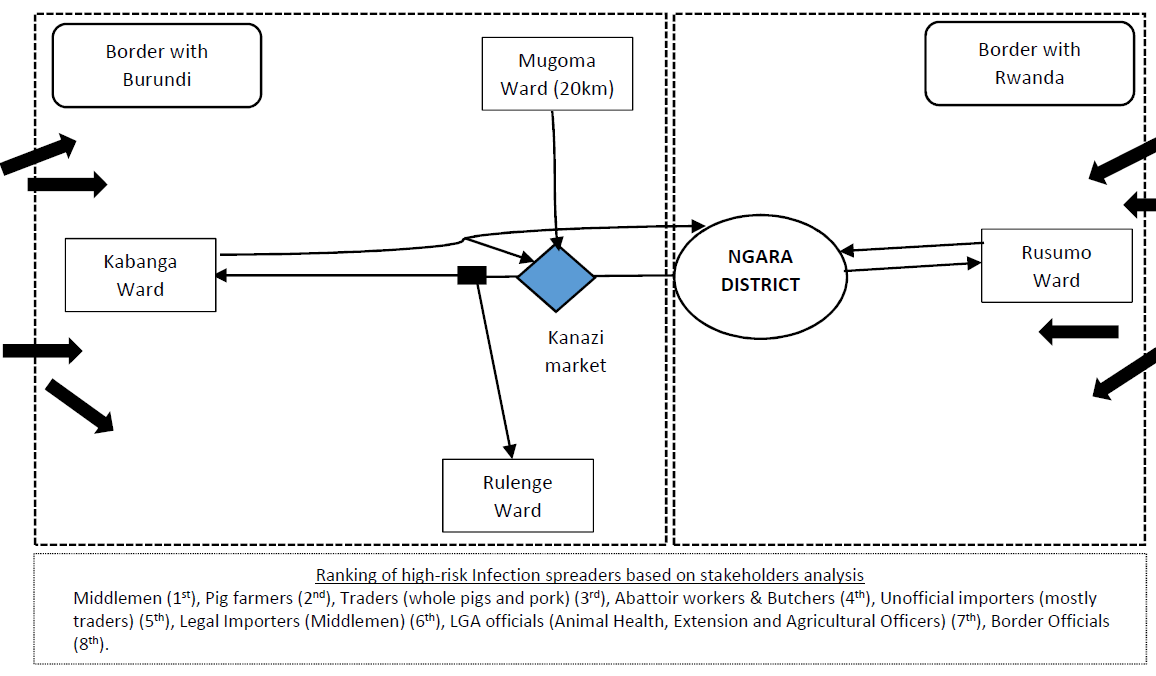
*

Supplementary Figure 5**: Schematic representation of intra-district movement of pig and pig products**


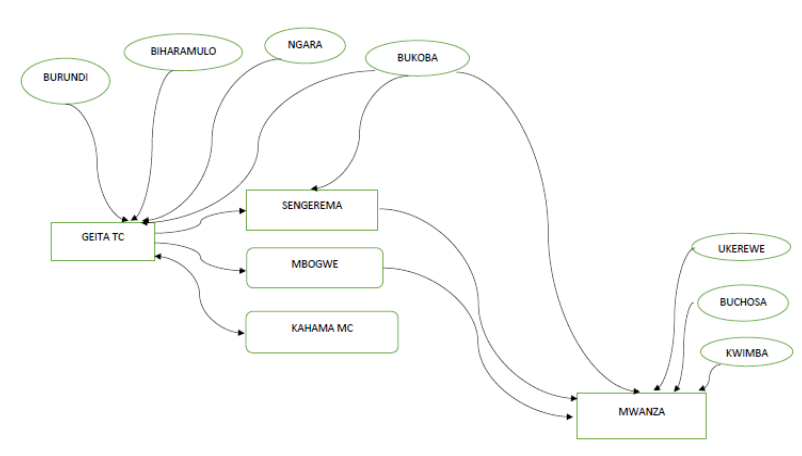


Supplementary Figure 6: Sample of pig movements to and from the farms and towards the slaughter slabs and livestock markets.
